# A micro X-ray computed tomography dataset of South African hermit crabs (Crustacea: Decapoda: Anomura: Paguroidea) containing scans of two rare specimens and three recently described species

**DOI:** 10.1093/gigascience/giy022

**Published:** 2018-03-14

**Authors:** Jannes Landschoff, Anton Du Plessis, Charles L Griffiths

**Affiliations:** 1 Department of Biological Sciences and Marine Research Institute, University of Cape Town, Rondebosch, South Africa; 2 CT Scanner, Central Analytical Facility, Stellenbosch University, Stellenbosch, South Africa

**Keywords:** micro-CT, μCT, nano-CT, 3D, cybertype, e-type, Diogenidae, Paguridae, Parapaguridae, taxonomy, deep sea species

## Abstract

**Background:**

Along with the conventional deposition of physical types at natural history museums, the deposition of 3-dimensional (3D) image data has been proposed for rare and valuable museum specimens, such as irreplaceable type material.

**Findings:**

Micro computed tomography (μCT) scan data of 5 hermit crab species from South Africa, including rare specimens and type material, depicted main identification characteristics of calcified body parts. However, low-image contrasts, especially in larger (>50 mm total length) specimens, did not allow sufficient 3D reconstructions of weakly calcified and fine characteristics, such as soft tissue of the pleon, mouthparts, gills, and setation. Reconstructions of soft tissue were sometimes possible, depending on individual sample and scanning characteristics. The raw data of seven scans are publicly available for download from the GigaDB repository.

**Conclusions:**

Calcified body parts visualized from μCT data can aid taxonomic validation and provide additional, virtual deposition of rare specimens. The use of a nondestructive, nonstaining μCT approach for taxonomy, reconstructions of soft tissue structures, microscopic spines, and setae depend on species characteristics. Constrained to these limitations, the presented dataset can be used for future morphological studies. However, our virtual specimens will be most valuable to taxonomists who can download a digital avatar for 3D examination. Simultaneously, in the event of physical damage to or loss of the original physical specimen, this dataset serves as a vital insurance policy.

## Data Description

### Motivation and background

Micro X-ray computed tomography (μCT) is an emerging tool in taxonomy [1]. In addition to being one of the most powerful methods for producing multidimensional scientific images, another benefit is the creation of a 3-dimensional (3D) dataset, also referred to as a “cybertype,” that not only acts as a duplicate of the physical museum types but can much more easily be digitally stored and distributed. The increasing availability of μCT facilities, computing power, and online data repositories is stimulating the use of virtual types, including cybertypes [2, 3].

With more than 1100 species worldwide, paguroid hermit crabs form the largest group of anomuran decapod crustaceans [4]. Hermit crabs primarily inhabit empty gastropod shells. Their overall body plan has become modified to suit this specialized habitat. Body proportions have had to remain within certain dimensional limitations, while the pleon and most of the carapace have become soft, flexible, and generally coiled so that they can be retracted into the spiral of the shell. Having a conservative and generally similar body plan, as well as having half of their body parts membranous and, for the most part, lacking identification characteristics, hermit crabs often remain taxonomically poorly understood. Correct identification requires careful examinations and depends heavily on the quality of the original species descriptions and illustrations. Until now, literature-based descriptions have proven inadequate, and the only option has been to loan and examine physical museum material. However, loaning such material from globally spread natural history museums is not only costly but also time consuming. Permission to loan material may also be refused, especially where type specimens are involved.

The dataset presented in this study was primarily created to visually support descriptive taxonomic studies of hermit crabs [5, 6]. However, the 3D raw data that are publicly made available here can also be used for morphological comparisons, including species validations, without having to examine the physical specimen. To our knowledge, this is the first publicly available 3D μCT dataset of hermit crabs and also of decapod crustaceans. It includes scans of three recently described species and two scans of rare species, one of which is from a deep sea habitat at >500 m depths. While the inspection of virtual representations of a specimen does not entirely replace the examination of a physical sample, the dataset presented here will serve as a taxonomic tool that may be sufficient to confirm species identification and that can be consulted before the physical material has to be sourced from natural history collections. By making this μCT dataset publicly available, we provide taxonomists potentially more timely and cost-efficient options for specimen examination and character comparisons.

### Sampling and specimens scanned

Scans of seven specimens of five species belonging to three families of hermit crabs (*Paguroidea sensu*; McLaughlin 2003) are presented: *Diogenes albimanus* Landschoff and Rahayu, 2018 and *Cancellus macrothrix* Stebbing, 1924 (Family Diogenidae); *Pagurus* sp. Landschoff and Komai, (in prep). and *Goreopagurus poorei* Lemaitre and McLaughlin, 2003 (Family Paguridae); and *Paragiopagurus atkinsonae* Landschoff and Lemaitre, 2017 (Family Parapaguridae). All specimens were collected in South Africa during various sampling operations. Physical specimens used in this study are deposited at the Iziko South African Museum (SAMC), Cape Town, and at the National Museum of Natural History (USNH), Smithsonian Institution, Washington, DC, USA. Detailed specimen information can be found in Table 1, where the standard size measurement for hermit crabs is given as shield length (in millimeters), measured from the tip of the rostrum to the midpoint of the posterior margin of the shield. To give a better understanding of the overall size dimensions, “total length,” as given in the text, refers to the length of a specimen when it is stretched out and measured from the distal-most tip of the respectively longer cheliped to the outer edge of the curvature of the pleon.

**Table 1:** Scanning details of micro-CT dataset of South African hermit crabs.

Species	Museum ID	Sex and size (shield length)	Preservative, scanning medium	Isotropic voxel size (μm)	Voltage (kV)/current (μA)/filter	Name of scan
*Diogenes albimanus*	SAMC MB-A066353 (holotype)	Ovig. female (2.0 mm)	96% EtOH, wrapped in parafilm	6	100/240/none	Diogenes_albimanus_f_holotype
*Pagurus* sp.	SAMC MB-A066790 (holotype)	Male (2.7 mm)	96% EtOH, in solution	11	60/310/none	Pagurus_sp_m_holotype
				4.5	60/310/none	Pagurus_sp_m_holotype
	SAMC MB-A066770 (paratype)	Ovig. female (2.4 mm)	96% EtOH, wrapped in parafilm	5	60/240/none	Pagurus_sp_f_paratype
*Paragiopagurus atkinsonae*	USNH 1 292 083 (holotype)	Male (7.0 mm)	Fresh, in air	20	100/100/none	Paragiopagurus_atkinsonae_m_ holotype
	SAMC MB-A066812 (paratype)	Female (7. 3 mm, in zoanthid shell)	Fresh, in air	20	120/240/0.1mm Cu	Paragiopagurus_atkinsonae_f paratype
*Cancellus macrothrix*	SAMC MB-A066204	Male/female (9.0 mm)	96% EtOH, in solution	20.4	100/100/none	Cancellus_macrothrix
*Goreopagurus poorei*	USNM 1 292 090	Male (4.5 mm)	Fresh, in air	35	100/100/none	Goreopagurus_poorei_m

Specimens of *Pagurus* sp. are denoted as holo- and paratype, but this status is pending the acceptance of the publication in which this species is officially described.

Specimens of *D. albimanus* and *Pagurus* sp. were collected on 14–15 October 2015 during a scuba dive at 20 m depth off Pumula (GPS S30°38.34’, E30°32.94’) and Hibberdene (GPS S30°34.92’, E30°34.86’), respectively, on the southern coast of KwaZulu-Natal. Both species are small reef inhabitants with a total length of about 20 mm. All samples were preserved in 96% ethanol. Although in good condition, the left cheliped of the male of *Pagurus* sp. had broken off but was still present in the sample (this specimen will be the selected holotype pending acceptance of the publication that describes this new species; specimens of *Pagurus* sp. are from hereon referred to as holo- and paratype). The specimen of *C. macrothrix* was collected on 13 May 2015 during a 20-m scuba dive near Roman Rock in False Bay (GPS S34°11.16’, E18°25.63’) and also preserved in 96% ethanol. The specimen is unusual in that it has both male and female gonopores on the coxae of the third and fifth pereopods. Due to previous tissue extraction for DNA bar coding, the scan is missing the 5 distal-most segments of the second left pereopod, but the 3 distal-most segments are still present in the physical sample. Specimens of *G. poorei* and *P. atkinsonae* were collected during research cruises conducted by the South African Department of Forestry and Fisheries and were frozen onboard. The specimen of *G. poorei* was trawled on 15 October 2016 from 520 m at the shelf edge of the Agulhas Bank on the south coast (GPS S35°14.94’, E22°50.82’). This sampling event constituted the first record of *G. poorei* in South Africa and represents a remarkable range extension, as this species had previously been recorded only from Tasmania [7]. *Paragiopagurus atkinsonae* were trawled on 11 March 2016 from two nearby sampling stations on the west coast (male holotype from 265 m, GPS S31°52.80’, E16°57.12’; female paratype from 199 m, GPS S32°22.98’, E17°27.78’). The female paratype was left in its original shell, which is a carcinoecium created by a mutualistic species of zoanthid (probably *Epizoanthus* spp.). *Cancellus macrothrix*, *P. atkinsonae*, and *G. poorei* are all medium-sized hermit crabs of about 50–70 mm total length.

### Scanning and quality control

Using several methods of sample preparation, all specimens were scanned using two systems at the CT Scanner Facility at Stellenbosch University, South Africa [8]. The male holotype of *P. atkinsonae* and the specimen of *G. poorei* were defrosted, mounted on top of a plastic rod with dense polystyrene foam as a platform, and scanned fresh at 20 μm and 35 μm isotropic voxel resolution, respectively, using a General Electric Phoenix V|Tome |X L240 with NF180 option. The same method was used for the paratypic female *P. atkinsonae* left in its carcinoecium shell, which was scanned at 20 μm isotropic voxel resolution. However, as in the remaining scans listed below, this scan was performed using a General Electric Phoenix Nanotom S. For the scans of the holotype of *Pagurus* sp. and of *C. macrothrix*, each specimen was placed in a small plastic container filled with ethanol, in which the samples were supported by dense polystyrene foam. The containers were then mounted on a plastic rod using double-sided tape and placed whole in the scanner. *Cancellus macrothrix* was scanned at 20.4 μm isotropic voxel resolution; the holotype of *Pagurus* sp. was scanned in two parts. Because the left cheliped had broken off during previous handling of the sample, the whole animal was scanned at 11 μm isotropic voxel resolution, while the individual scan of the left cheliped allowed for an isotropic voxel resolution of 4.5 μm, which resulted in the highest resolution scan of this dataset. As a last and slightly different method, the ovigerous female holotype of *D. albimanus* and the ovigerous female paratype of *Pagurus* sp. were taken out of ethanol, wrapped in parafilm (Bemis NA, Neenah, WI, USA), and mounted on rigid foam that was glued to the top of a plastic rod. They were subsequently scanned at 6 μm and 5 μm isotropic voxel resolution, respectively.

Parameter optimization for all scans performed followed the method of du Plessis et al. [9] and included settings of X-ray spot sizes to not exceed the selected scan resolution, as well as good X-ray penetration indicated by high transmitted brightness values in the live digital X-ray images. For the small species *D. albimanus* and *Pagurus* sp., the scan parameters were set at 60 kV and 240 μA or 310 μA, and no filter was used. The parameters for the larger specimens of *P. atkinsonae*, *G. poorei*, and *C. macrothrix* were set at 100 kV and 100 μA, and no filter was used for the scans of the hermit crabs only. However, in order to allow for sufficient X-ray penetration through the carcinoecium shell, the female paratype of *P. atkinsonae* was scanned at a higher voltage (120 kV) and current (240 μA), using a 0.1-mm copper beam filter to reduce potential beam hardening artifacts. These scan parameters are summarized in Table 1. Background detector calibrations before each scan, as well as visual inspections of the reconstruction images, ensured high data quality and good image contrast. Image acquisition in all scans was between 333 and 500 ms per image, with average 1 and skip 1, as well as an activated detector shift to minimize ring artifacts. Between 1600 and 3600 images were recorded in steps during one full sample rotation. Reconstructions of the acquired projection images were computed using the system supplied General Electric Datos software and were consequently analyzed using Volume Graphics VGStudio Max 3.0. (Heidelberg, Germany). One novel aspect of these data is the combination of scans of parts of the holotype of *Pagurus* sp., which were aligned and overlaid using the *merge volumes* function in VGStudio Max. The merged volume can therefore be downloaded as a single combined dataset.

### Data quality and limitations

**Figure 1:**
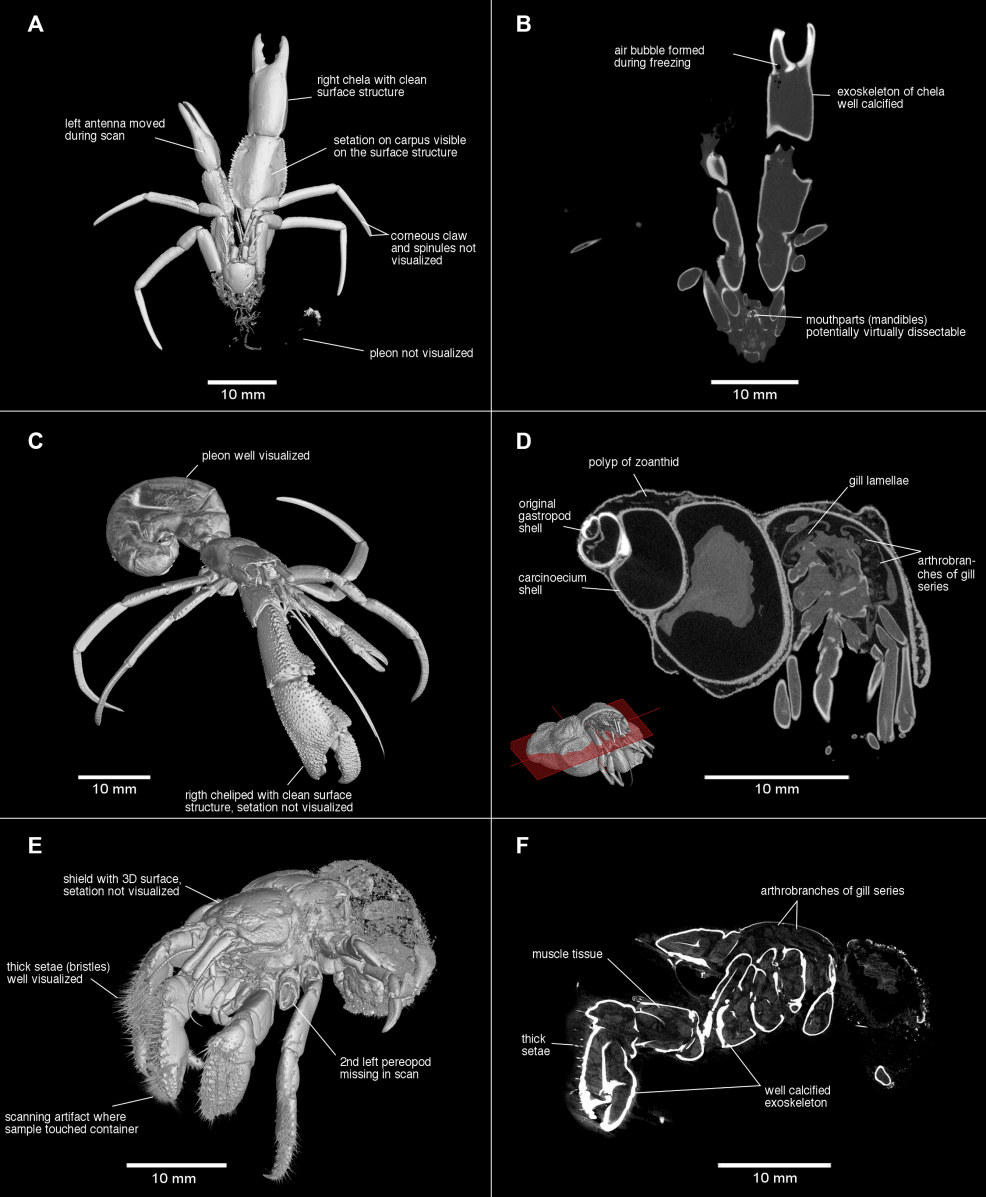
Micro-CT scanning images (2-dimensional) and combination of surface and volume reconstructions (3-dimensional) of medium-sized hermit crabs (50–70 mm total length). A and B) *Goreopagurus poorei*, male 4.5 mm shield length (SL) (USNM 1 292 090); C) *Paragiopagurus atkinsonae*, male holotype 7.0 mm SL (USNH 1 292 083); D) *Paragiopagurus atkinsonae*, female paratype (in carcinoecium shell, SAMC MB-A066812); E and F) *Cancellus macrothrix*, male/female 9.0 mm SL (SAMC MB-A066204).

Scan quality varied based on the resolution of the scans, size of the specimen, species characteristics, and sample preparation. The scans of the larger species of *G. poorei* and *P. atkinsonae* showed major morphological structures but did not reveal enough resolution to study fine details such as the setation or corneous spinulation (Fig. 1A–D) because, for larger samples, a wider field of view invariably compromises resolution. Hermit crabs are also a challenging taxon to study using μCT scanning, as a vast proportion of the body consists of soft tissue. With the lowest resolution of the presented scans, the data for *G. poorei* were only usable for visualizations of the well-calcified areas of the exoskeleton such as the chelipeds (Fig. 1A and B). Also, although the sample was fresh and not preserved in ethanol, the left second antenna moved slightly during the scanning process as the sample was drying (Fig. 1A). We found that scanning specimens (particularly larger ones) in an airtight container to prevent them from drying out exacerbated the problem of having to move the sample further away from the X-ray source of the scanner, causing significant loss of resolution. Therefore, because hermit crabs have many joints and flexible soft parts that are prone to movements, the better method was to keep the scanning time short when the fresh samples were scanned while exposed to air.

**Figure 2:**
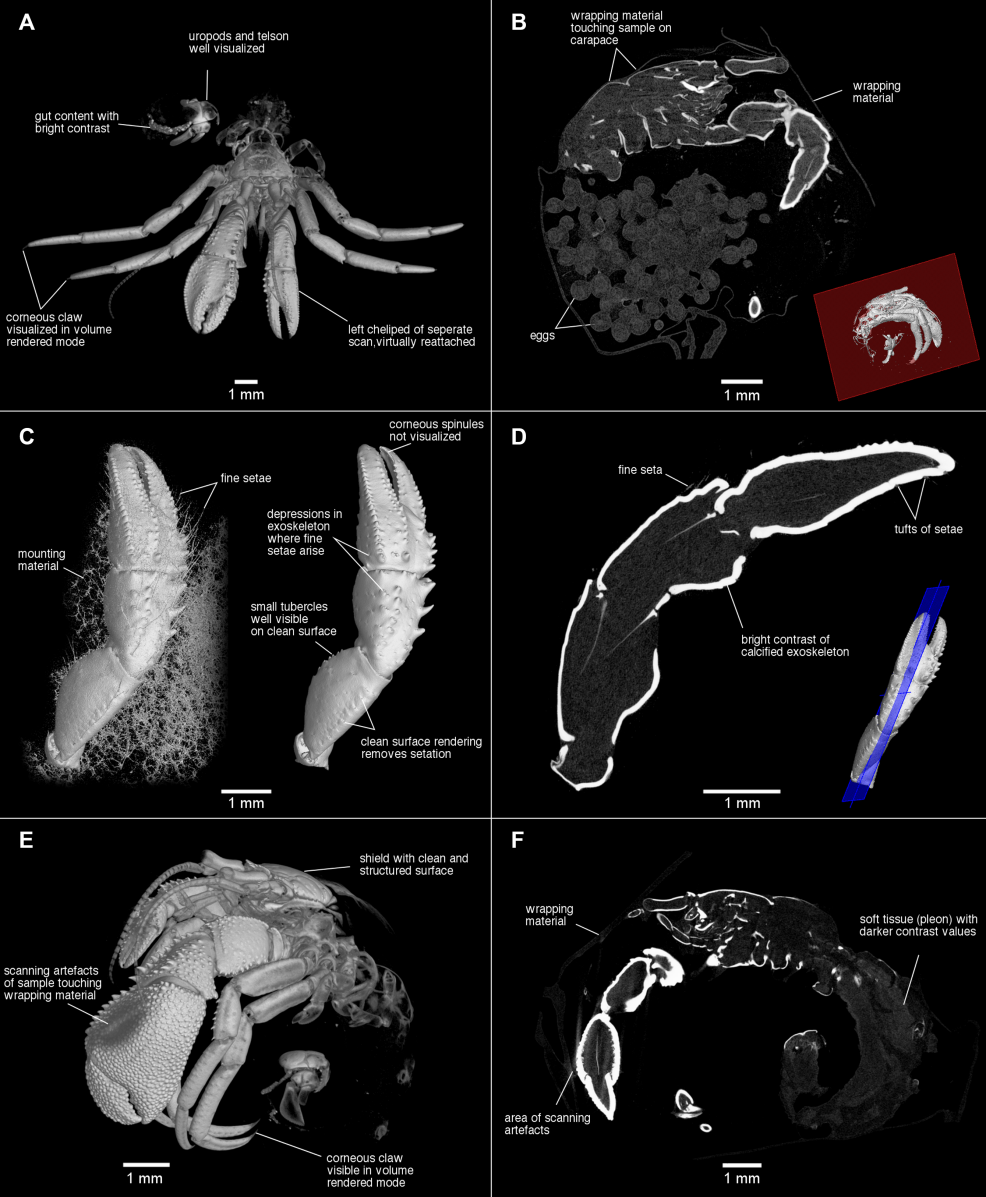
Micro-CT scanning images (2-dimensional) and combination of surface and volume reconstructions (3-dimensional) of small-sized hermit crabs (20 mm total length). A) *Pagurus* sp., male holotype 2.7 mm shield length (SL) (SAMC MB-A066790); B) *Pagurus* sp., ovigerous female paratype 2.4 mm SL (SAMC MB-A066770); C and D) left cheliped of same as A; E and F *Diogenes albimanus*, ovigerous female paratype 2.0 mm SL (SAMC MB-A066353).

The scans of *P. atkinsonae* are of better quality than the scan of *G. poorei*; both were scanned with settings to keep the scanning time to less than 30 min. The quality of the latter allowed visualization of some soft tissue such as the pleon (Fig. 1C); some information on the gills is retrievable from the scan of the female (Fig. 1D). Further overcoming the problem of drying samples and sample movements during the scanning process, scanning the holotype of *Pagurus* sp. in ethanol resulted in a clean, high-resolution surface scan (Fig. 2A). Nevertheless, the decreased density difference between the sample and the surrounding medium hindered detection of fine, soft structures. It particularly “removed” all setation, although some setae are visible in the highest resolution scan of the left cheliped, but only if the brightness contrast threshold in the 3D rendering is set very low. However, it is almost impossible to separate the sample from “noise” caused by the mounting material and to get a clean image (Fig. 2C).

Because the long-term effects of staining agents on tissue remain unknown [10], we refrained from contrast-enhancement techniques for these rare or type specimen scans. Moreover, a test scan that used iodine as the staining agent did not result in markedly better image contrast on taxonomically important features (scan not included in this dataset). Second, after sample preparation and the iodine-staining test scan, we noticed that the eggs of the stained and scanned ovigerous female started to fall off the pleopods more easily. Although setation was difficult to visualize in the 2 scans of the holotype of *Pagurus* sp., the scan of the left cheliped, in particular, reveals exceptional detail. For example, it is possible to detect the exact position of each seta as a depression from which each seta arises on the exoskeleton surface (Fig. 2D and C). Combined, the 2 scans of this holotypic specimen show great detail but predominantly of calcified body parts only. Scanned in the same way while being submerged in ethanol, the scan data of *C. macrothrix* are of high quality as well. In contrast to the in-ethanol scans of *Pagurus* sp., they also reveal a number of details that include soft tissue or fine structures. For example, the very thick setae, as alluded to in the species name of *C. macrothrix*, are visible (Fig. 1E and F), highlighting that visualizations of soft tissue depend on sample characteristics.

Overall, the two scans (holotype *D. albimanus* and female paratype *Pagurus* sp.) that were performed with the samples wrapped in parafilm potentially show the most detail of all scans (Fig. 2B, E, and F). The samples were not placed in a container and could be mounted extremely close to the X-ray source of the scanner, which significantly improved resolution of the scans. From these two scans it is possible to retrieve information on many soft tissue parts, including the pleon and eggs attached to the pleopods, small corneous spinules, and setation. However, the quality of the data is still not sufficient, e.g., to study the taxonomically highly important gills. Furthermore, this sample preparation had a disadvantage in that wherever parafilm touched the specimen, unwanted scanning surface artifacts were created that are difficult to eliminate in the visualizations. Scanning artifacts that result from wrapping material can, e.g., be found around the shield of the female *Pagurus* sp. (Fig. 2B), as well as on the left cheliped of *D. albimanus* (Fig. 2E and F) and also in the scan of *C. macrothrix*, which was scanned in a small container in ethanol (Fig. 1E and F). Furthermore, in the scan of *D. albimanus*, the parafilm also increased beam-hardening effects on the edge of the scan and ventrally of the specimen, but these are easily removed in the visualization software. Last, the female paratype of *Pagurus* sp. has a slightly broken right cheliped, which might have been damaged during collection. The cracks in the exoskeleton of the carpus are visible in the scan (not pictured in the figures) and are not a result of the scanning but rather derived from the damaged sample. However, all major specimen characteristics can still be studied in great detail.

In conclusion, the quality of each scan in this dataset varied and is dependent on sample characteristics and scanning protocol. Due to a lower resolution from a wider field of view, the scans of the larger specimens show good surface details of the calcified body parts but insufficiently depict information on small features. In contrast, the scans of the smaller specimens show better details, including some soft tissue, mainly because their small size allowed scanning at a much higher resolution. Protocol optimization for future studies includes use of the smallest possible container in which the sample can be placed (in air, but without touching the container and mounting material) and that can be mounted as close to the X-ray sources of the scanner as possible. However, in this study, a good compromise was found that allowed high scanning quality while keeping the effort of data collection reasonable.

### Re-use potential

While the dataset described here can be used for morphological studies in general, any such research attempt would lie within the limitation of using information derived from the calcified parts of the specimens. As mentioned above, the scans of the larger specimens of *G. poorei* and *P. atkinsonae* are too low in resolution to include analyses of soft tissue. The female paratype of *P. atkinsonae* is located in a carcinoecium shell of an anthozoan species (probably *Epizoanthus* spp.) that would have to be virtually removed prior to analysis. However, the scan can be used to study the zoanthid shell itself, which remains a poorly known structure. Some soft tissue information will be retrievable from the higher resolution scans of the *D. albimanus* and *Pagurus* sp. specimens that allowed a narrow field of view of the scanner, as well as of the medium-sized *C. macrothix*. This dataset was not designed for the analyses of internal anatomy and contains no or little information on internal organs. Instead, the value of the presented scans lies in the potential to download a 3D virtual copy of museum specimens that otherwise would have to be loaned. Shipment of specimens involves significant cost and effort, as well as the potential risk of damage or even complete loss of a specimen, while this dataset is freely available for download and can be examined by an unlimited number of people simultaneously. At the same time, it serves as an insurance policy should the original specimens ever get damaged or lost.

Using this dataset, researchers who want to validate a species and examine the specimens for comparison to other taxa are provided with a 3D, virtual, interactive view that makes it possible to derive information of some soft tissue and a suite of calcified characteristics. These are mainly the shield and cephalic appendages, the chelipeds, the pereopods, as well as the uropods and the telson. Experts on hermit crab taxonomy might object to the absence of information on important soft structures, such as the gills. Nevertheless, even if the scans do not show all of the important characteristics that are currently used in hermit crab taxonomy, they do show many characteristics such as the 3D shape of the chelipeds and pereopods in an exceptional way. Furthermore, the digital third dimension allows for internal character examination, even of type material.

## Supplementary Material

GIGA-D-17-00200_Original_Submission.pdf

GIGA-D-17-00200_Revision_1.pdf

GIGA-D-17-00200_Revision_2.pdf

Response_to_Reviewer_Comments_Original_Submission.pdf

Response_to_Reviewer_Comments_Revision_1.pdf

Reviewer_1_Report_(Original_Submission) -- Chris Armit 01 Sep 2017 Reviewed

Reviewer_2_Original_Submission_(Attachment)_GIGA-D-17-00200_review.pdf

Reviewer_2_Report_(Original_Submission) -- Stephanie Köhnk 10 Sep 2017 Reviewed

## Availability of supporting data

The presented dataset is deposited in the GigaScience Database repository [11]. In a previously published dataset on brittle stars, which is to date the only other available μCT dataset of South African invertebrates, we provided the full raw data as X-ray projection images from the scanner [12]. We now realize that users would not use the projections from the scanner and that these data make the dataset unnecessarily large. Therefore, for the dataset presented here, each scan contains the reconstructed stack slice images available as 16-bit tiff and, to compress the file sizes further, as 8-bit jpg stacks. The stack slice images comprise the volume data of the region of interest and can be read by any 3D visualization software package; slice images can also simply be viewed in any image viewer program. In addition, to provide an easy and rapid overview, each scan folder contains a 2D and 3D rendered image of each scan and a 3D, interactive STL file that can be read using most modern desktop computer software. These low-resolution 3D files are also viewable in the browser and hence give users a direct summary of the data.

## Abbreviations

3D: three dimensional; μCT: micro-computed tomography; CT: computed tomography; SAMC: Iziko South African Museum; SL: shield length; USNH: US National Museum of Natural History.

## Authors’ information

JL is a PhD student at the University of Cape Town. Under the supervision of CLG he studies the biodiversity of hermit crabs in South Africa, but has a broad interest in marine invertebrates and in crustacean biology in particular.

As a trained physicist, ADP manages the CT Scanner section of the Central Analytical Facility, Stellenbosch University. His broad research interest includes the biological applications of CT scanning and bio-inspired engineering.

CLG is Emeritus Professor at the University of Cape Town. His main current research interest is the documentation of South African marine biodiversity.

## References

[1] Faulwetter S, Dailianis T, Vasileiadou K Can micro-CT become an essential tool for the 21st century taxonomist? An evaluation using marine polychaetes. Microsc Anal 2014;11:9–12.

[2] Akkari N, Enghoff H, Metscher BD A new dimension in documenting new species: high-detail imaging for myriapod taxonomy and first 3D cybertype of a new millipede species (Diplopoda, Julida, Julidae). PLoS One 2015;e0135243:1–25. 10.1371/journal.pone.0135243 PMC4550252 26309113

[3] Garcia FH, Fischer G, Liu C X-ray microtomography for ant taxonomy: an exploration and case study with two new Terataner (Hymenoptera, Formicidae, Myrmicinae) species from Madagascar. PLoS One 2017;12:e0172641. 28328931 10.1371/journal.pone.0172641 PMC5362212

[4] McLaughlin PA, Komai T, Lemaitre R Annotated checklist of anomuran decapod crustaceans of the world (exclusive of the Kiwaoidae and families Chirostylidae and Galatheidae of the Galatheoidae) Part I—Lithodoidea, Lomisoidea and Paguroidea. Raffles Bull Zool 2010;23:5–107.

[5] Landschoff J, Lemaitre R Differentiation of three common deep-water hermit crabs (Crustacea, Decapoda, Anomura, Parapaguridae) from the South African demersal abundance surveys, including the description of a new species of *Paragiopagurus* Lemaitre, 1996. Zookeys 2017;676:21–45. 10.3897/zookeys.676.12987 PMC5523205 28769685

[6] Landschoff J, Rahayu DL A new species of the hermit crab genus *Diogenes* (Crustacea: Decapoda: Diogenidae) from the coast of KwaZulu-Natal, South Africa. Zootaxa 2018;4379:268–78. 10.11646/zootaxa.4379.2.7 29689988

[7] Landschoff J, Lemaitre R Crossing the Indian Ocean: a range extension for *Goreopagurus poorei* Lemaitre & McLaughlin, 2003 (Crustacea: Decapoda: Paguridae). Zootaxa 2017;4306:271–8.

[8] du Plessis A, le Roux S, Guelpa A The CT Scanner Facility at Stellenbosch University: an open access X-ray computed tomography laboratory. Nucl Instrum Methods Phys Res, Sect B 2016;384:42–49.

[9] du Plessis A, Broeckhoven C, Guelpa A Laboratory X-ray micro-computed tomography: a user guideline for biological samples. Gigascience 2017;6:1–11. 10.1093/gigascience/gix027 PMC5449646 28419369

[10] Faulwetter S, Vasileiadou A, Kouratoras M Micro-computed tomography: introducing new dimensions to taxonomy. Zookeys 2013;263:1–45. 10.3897/zookeys.263.4261 PMC3591762 23653515

[11] Landschoff J, Du Plessis A, Griffiths CL Supporting data for “a micro X-ray computed tomography dataset of South African hermit crabs (Crustacea: Decapoda: Anomura: Paguroidea)” GigaScience Database 2018 http://dx.doi.org/10.5524/100364. 10.1093/gigascience/giy022 PMC5890486 29635298

[12] Landschoff J, Du Plessis A, Griffiths CL A dataset describing brooding in three species of South African brittle stars, comprising seven high-resolution, micro X-ray computed tomography scans. Gigascience 2015;4:52. 26579220 10.1186/s13742-015-0093-2 PMC4647331

