# Erratum to: A micro X-ray computed tomography dataset of South African hermit crabs (Crustacea: Decapoda: Anomura: Paguroidea) containing scans of two rare specimens and three recently described species

**DOI:** 10.1093/gigascience/giz032

**Published:** 2019-03-14

**Authors:** Jannes Landschoff, Anton Du Plessis, Charles L Griffiths

**Affiliations:** 1 Department of Biological Sciences and Marine Research Institute, University of Cape Town, Rondebosch, South Africa; 2 CT Scanner, Central Analytical Facility, Stellenbosch University, Stellenbosch, South Africa

## Correction

In the final publication of “A micro X-ray computed tomography dataset of South African hermit crabs (Crustacea: Decapoda: Anomura: Paguroidea) containing scans of two rare specimens and three recently described species,” by Jannes Landschoff et al., many copyediting errors were erroneously published. These errors have been corrected throughout the article to improve clarity. No substantial revisions have been made to the paper's research.

The Publisher regrets this error.

