# Supplementary material for: A micro X-ray computed tomography dataset of South African hermit crabs (Crustacea: Decapoda: Anomura: Paguroidea) containing scans of two rare specimens and three recently described species
Source: Gigascience. 2018 Mar 14;7(4):giy022. doi: 10.1093/gigascience/giy022 (PMC5890486; doi:10.1093/gigascience/giy022)

GigaScience

A micro X-ray computed tomography dataset of South African hermit crabs (Crustacea: Decapoda: Anomura: Paguroidea), containing scans of two rare specimens and of three recently described species

--Manuscript Draft--

**Manuscript Number:**	GIGA-D-17-00200
**Full Title:**	A micro X-ray computed tomography dataset of South African hermit crabs (Crustacea: Decapoda: Anomura: Paguroidea), containing scans of two rare specimens and of three recently described species
**Article Type:**	Data Note
**Funding Information:**	
**Abstract:**	Background. Along with the conventional deposition of physical types at natural history museums, the deposition of three-dimensional (3D) image data has been proposed for rare and valuable museum specimens such as irreplaceable type material.<br>Findings. Micro computed tomography (µCT) scan data of five hermit crab species from South Africa, representing two scans of two rare specimens, three holotypic specimens, and two of selected paratypes, depicted main identification characters of calcified body parts. However, low image contrasts, especially in larger (>50 mm total length) specimens did not allow sufficient 3D reconstructions of weakly calcified or fine characters such as soft tissue of the pleon, mouthparts, gills, or the setation. Reconstructions of soft tissue were sometimes possible depending on individual sample and scanning characteristics. The raw data of seven scans are publicly available for download from the GigaDB repository.<br>Conclusions. Calcified body parts visualized from µCT data can aid taxonomic validation and provide additional, virtual deposition of rare specimens. Using a non-destructive, non-staining µCT approach for taxonomy, reconstructions of soft tissue structures, microscopic spines and of setae depend on species characteristics. Constrained to these limitations, the presented dataset can be used for future morphological studies. However, our virtual specimens will be most valuable to taxonomists who can download a digital avatar for 3D examination. Simultaneously, in the possible event of damaging or even loosing a physical specimen, this dataset serves as an insurance policy.
**Corresponding Author:**	Jannes Landschoff<br><br>SOUTH AFRICA
**Corresponding Author Secondary Information:**	
**Corresponding Author's Institution:**	
**Corresponding Author's Secondary Institution:**	
**First Author:**	Jannes Landschoff
**First Author Secondary Information:**	
**Order of Authors:**	Jannes Landschoff
	Anton Du Plessis
	Charles L Griffiths
**Order of Authors Secondary Information:**	
**Opposed Reviewers:**	
**Additional Information:**	
**Question**	**Response**
Are you submitting this manuscript to a	No

Powered by Editorial Manager® and ProduXion Manager® from Aries Systems Corporation

special series or article collection?	
**Experimental design and statistics**<br><br>Full details of the experimental design and statistical methods used should be given in the Methods section, as detailed in our Minimum Standards Reporting Checklist. Information essential to interpreting the data presented should be made available in the figure legends.<br><br>Have you included all the information requested in your manuscript?	Yes
**Resources**<br><br>A description of all resources used, including antibodies, cell lines, animals and software tools, with enough information to allow them to be uniquely identified, should be included in the Methods section. Authors are strongly encouraged to cite Research Resource Identifiers (RRIDs) for antibodies, model organisms and tools, where possible.<br><br>Have you included the information requested as detailed in our Minimum Standards Reporting Checklist?	Yes
**Availability of data and materials**<br><br>All datasets and code on which the conclusions of the paper rely must be either included in your submission or deposited in publicly available repositories (where available and ethically appropriate), referencing such data using a unique identifier in the references and in the "Availability of Data and Materials" section of your manuscript.<br><br>Have you have met the above requirement as detailed in our Minimum Standards Reporting Checklist?	Yes

Manuscript

Click here to download Manuscript
DataNote_hermit_crabs_Submission_7Aug2017.docx

Data Note

A micro X-ray computed tomography dataset of South African hermit crabs (Crustacea: Decapoda: Anomura: Paguroidea), containing scans of two rare specimens and three recently-described species

Authors:

Jannes Landschoff[1]*, Anton Du Plessis[2] and Charles L. Griffiths[1]

Authors details:

[1]Department of Biological Sciences and Marine Research Institute, University of Cape Town, Rondebosch, South Africa.

[2]CT Scanner, Central Analytical Facility, Stellenbosch University, Stellenbosch, South Africa.

*corresponding author: jannes@landschoff.net

Abstract

Background. Along with the conventional deposition of physical types at natural history museums, the deposition of three-dimensional (3D) image data has been proposed for rare and valuable museum specimens, such as irreplaceable type material.

Findings. Micro computed tomography (µCT) scan data of five hermit crab species from South Africa, two of rare specimens, three of holotypic specimens, and two of selected paratypes, depicted main identification characters of calcified body parts. However, low image contrasts, especially in larger (>50 mm total length) specimens did not allow sufficient 3D reconstructions of weakly-calcified or fine characters, such as soft tissue of the pleon, mouthparts, gills, or of the setation. Reconstructions of soft tissue were sometimes possible, depending on individual sample and scanning characteristics. The raw data of seven scans are publicly available for download from the GigaDB repository.

Conclusions. Calcified body parts visualized from µCT data can aid taxonomic validation and provide additional, virtual deposition of rare specimens. Using a non-destructive, non-staining µCT approach for taxonomy, reconstructions of soft tissue structures, microscopic spines and of setae depend on species characteristics. Constrained to these limitations, the presented dataset can be used for future morphological studies. However, our virtual specimens will be most valuable to taxonomists who can download a digital avatar for 3D examination. Simultaneously, in the possible event of physical damage to, or loss of, the original physical specimen, this dataset serves as a vital insurance policy.

Keywords: microCT, µCT, nanoCT, 3D, cybertype, e-type, Diogenidae, Paguridae, Parapaguridae, taxonomy, deep sea species

Data description

Motivation and background:

Micro X-ray computed tomography (µCT) is an emerging tool in taxonomy [1]. Besides being one of the most powerful methods to produce multidimensional scientific images, another benefit is the creation of a three-dimensional (3D) dataset also referred to as ‘cybertype’, which not only acts as a duplicate of the physical museum types, but can much more easily be digitally stored and distributed. Due to the growing availability of µCT facilities, computing power, and online data repositories, virtual types, including cybertypes, are being used with increasing frequently [2,3].

With over 1,100 species worldwide, paguroid hermit crabs form the largest group of anomuran decapod crustaceans [4]. Hermit crabs mostly inhabit empty gastropod shells and their overall body plan has become modified to suit this specialised habitat. Body proportions have had to remain within certain dimensional limitations, while the pleon and most of the carapace have become soft, flexible and generally coiled such that they can be retracted into the spiral of the shell. Having a conservative and generally similar body plan, as well as having half the body parts membranous and for the most lacking identification characters, hermit crabs often remain taxonomically poorly understood. Correct identifications require careful examinations, and, maybe even more than in other taxonomic groups, they depend heavily on the quality of the original species descriptions and illustrations. Until now, when literature-based descriptions have proven inadequate, the only option has been to loan and examine physical museum material. However, loaning such material from globally-spread natural history museums is not only costly, but also a time-consuming undertaking. Permission to loan material may also be refused, especially where type specimens are involved.

The dataset presented in this study was primarily created to visually support descriptive taxonomic studies of hermit crabs [5–7], but the 3D raw data that are publicly made available here, can also be used for morphological comparisons, including species validations, without examining the physical specimen. To our knowledge, this is the first publicly-available 3D µCT dataset of hermit crabs and

also of decapod crustaceans. It includes scans of the types of three recently described species and two scans of rare species, one of which is from a deep sea habitat at >500 m depths. While the inspection of virtual representations of a specimen does not entirely replace the examination of a physical museum voucher, the dataset presented here will serve as an extra taxonomic tool that may be sufficient to confirm species identification and that can be consulted before the physical material has to be sourced from natural history collections. Through making this μCT dataset publicly available, we thus provide taxonomists potentially more time and cost efficient options for specimen examination and character comparisons.

Sampling and specimens scanned

Scans of seven specimens of five species, belonging to three families of hermit crabs (Paguroidea *sensu* McLaughlin 2003) are presented: *Diogenes albimanus* Landschoff & Rahayu, [in prep.] and *Cancellus macrothrix* Stebbing, 1924 (Family Diogenidae), *Pagurus fraserorum* Landschoff & Komai, [in prep.] and *Goreopagurus poorei* Lemaitre & McLaughlin, 2003 (Family Paguridae), as well as *Paragiopagurus atkinsonae* Landschoff & Lemaitre, 2017 (Family Parapaguridae). All specimens were collected in South Africa during various sampling operations and physical specimens used in this study are deposited at the Iziko South African Museum (SAMC), Cape Town, South Africa, and at the National Museum of Natural History (USNH), Smithsonian Institution, Washinghton D.C., USA. Detailed specimen information can be found in Table 1., where the standard size measurement for hermit crabs is given as shield length (SL), measured from the tip of the rostrum to the midpoint of the posterior margin of the shield in millimetres. To give a better understanding of the overall size dimensions, 'total length' as given in the text refers to the length of a specimen when it is stretched out and measured from the distal-most tip of the respectively longer cheliped to the outer edge of the curvature of the pleon.

Specimens of *D. albimanus* and *P. fraserorum* were collected on 14-15 October 2015 during a Scuba dive at 20 m depth off Pumula (GPS S30°38.34', E30°32.94') and Hibberdene (GPS S30°34.92', E30°34.86'), respectively, on the southern coast of KwaZulu-Natal. Both these species are small reef inhabitants

with a total length of about 20 mm. All samples were preserved in 96% ethanol. Although in good condition, the left cheliped of the male holotype of *P. fraserorum* has broken off, but is still present in the sample. The specimen of *C. macrothrix* was collected on 13 May 2015 during a 20 m Scuba dive near Roman Rock in False Bay (GPS S34°11.16', E18°25.63'), and also preserved in 96% ethanol. The specimen is unusual in that it has both male and female gonopores on the coxae of the third and fifth pereopods. Due to previous tissue extraction for DNA barcoding the scan is missing the five distal-most segments of the second left pereopod, but the three distal-most segments are still present in the physical sample. Specimens of *G. poorei* and of *P. atkinsonae* were collected during research cruises conducted by the South African Department of Forestry and Fisheries (DAFF), where they were frozen onboard. The specimen of *G. poorei* was trawled on 15 October 2016 from 520 m at the shelf edge of the Agulhas Bank on the South coast (GPS S35°14.94', E22°50.82'). This sampling event constituted the first record of *G. poorei* in South Africa and represents a remarkable range extension, as this species had previously been recorded only from Tasmania [8]. *P. atkinsonae* were trawled on 11 March 2016 from two nearby sampling stations on the West coast (male holotype from 265 m, GPS S31°52.80', E16°57.12', female paratype from 199 m, GPS S32°22.98', E17°27.78'). The female paratype was left in its original shell, which is a carcinoecium created by a mutualistic species of zooanthid (probably *Epizoanthus* spp.). *Cancellus macrothrix*, *P. atkinsonae* and *G. poorei*, are all medium-sized hermit crabs of about 50-70 mm total lengths.

Table 1. Scanning details of micro CT dataset of South African hermit crabs.

Species	Museum ID	Sex & Size (shield length in mm)	Resolution (μm)	Voltage (kV)/ current (kV)/ filter	name of scan
Diogenes albimanus	SAMC MB-A066353 (holotype)	ovig. female (2.0)	6	100/240/none	Diogenes_albimanus_f_holotype
Pagurus fraserorum	SAMC MB-A066790 (holotype)	male (2.7)	11	60/310/none	Pagurus_fraserorum_m_holotype
			4.5	60/310/none	Pagurus_fraserorum_m_holotype
	SAMC MB-A066770 (paratype)	ovig. female (2.4)	5	60/240/none	Pagurus_fraserorum_f_paratype
Paragiopagurus atkinsonae	USNH 1292083 (holotype)	male (7.0 mm)	20	100/100/none	Paragiopagurus_atkinsonae_m_holotype
	SAMC MB-A066812	female (in zooanthid shell)	20	120/240/0.1mm Cu	Paragiopagurus_atkinsonae_f_paratype
Cancellus macrothrix	SAMC MB-A066204	male/female (9.0)	20.4	100/100/none	Cancellus_macrothrix

Goreopagurus poorei	USNM 1292090	male (4.5)	35	100/100/none	Goreopagurus_poorei_m

Scanning and quality control

Using several methods of sample preparation, all specimens were scanned using two systems at the CT Scanner Facility at Stellenbosch University, South Africa [9]. The male holotype of *P. atkinsonae,* as well as the specimen of *G. poorei,* were defrosted, mounted on top of a plastic rod with dense polystyrene foam as a platform, and scanned fresh at 20 µm and 35 µm isotropic voxel resolution, respectively, using a General Electric Phoenix V|Tome|X L240 with NF180 option. The same method was applied to the paratypic female *P. atkinsonae* left in its carcinoecium shell, which was scanned at 20 µm isotropic voxel resolution. However, like in all other remaining scans listed below, this scan was performed using a General Electric Phoenix Nanotom S. For the scans of the holotype of *P. fraserorum* and of *C. macrothrix,* specimens were each placed in a small plastic container filled with ethanol, in which the samples were supported by dense polystyrene foam. The containers were then mounted on a plastic rod using double-sided tape and placed in the scanner whole. *C. macrothrix* was scanned at 20.4 µm isotropic voxel resolution, whereas the holotype of *P. fraserorum* was scanned in two parts. Because the left cheliped had broken off during previous handling of the sample, the whole animal was scanned at 11 µm isotropic voxel resolution, while the individual scan of the left cheliped allowed for an isotropic voxel resolution of 4.5 µm, which resulted in the highest resolution scan of this dataset. As a last and slightly different method, the ovigerous female holotype of *D. albimanus* and the ovigerous female paratype of *P. fraserorum* were taken out of ethanol, wrapped in parafilm (Bemis NA, Neenah, WI, United States), and again mounted on rigid foam, which itself was glued to the top of a plastic rod. They were subsequently scanned at 6 µm and 5 µm isotropic voxel resolution, respectively.

Parameter optimization for all scans performed followed du Plessis et al. and included settings of X-ray spot sizes to not exceed the selected scan resolution, as well as good X-ray penetration [10], as indicated by high transmitted brightness values in the live digital X-ray images. For the small species *D. albimanus* and *P. fraserorum,* the scan parameters were set at 60 kV and 240 µA or 310 µA, using

no filter. The parameters for the larger specimens of *P. atkinsonae*, *G. poorei*, and *C. macrothrix* were set at 100 kV and 100 μA, and also used no filter for the scans of the hermit crabs only. However, in order to allow for sufficient x-ray penetration through the carcinoecium shell the female paratype of *P. atkinsonae* was scanned at a higher voltage (120kV) and current (240 μA), using a 0.1 mm copper beam filter to reduce potential beam hardening artefacts. Furthermore, background detector calibrations before each scan, as well as visual inspections of the reconstruction images, ensured high data quality and good image contrast. Image acquisition in all scans was between 333 and 500 ms per image, with average 1 and skip 1, as well as activated detector shift to minimize ring artefacts. Between 1600 and 3600 images were recorded in steps during one full sample rotation. Reconstructions of the acquired projection images were computed using the system supplied General Electric Datos software, and were consequently analysed using Volume Graphics VGStudio Max 3.0. (Heidelberg, Germany). One novel aspect of these data is the combination of scans of parts of the holotype of *P. fraserorum,* which were aligned and overlaid using the *merge volumes* function in VGStudio Max. The merged volume can therefore be downloaded as a single combined dataset.

Data quality and limitations

Scan quality varied, based on the resolution of the scans, size of the specimen scanned, but also on species characteristics and sample preparation. The scans of the larger species of *G. poorei* and *P. atkinsonae* showed major morphological structures, but did not reveal enough resolution to study fine details, such as the setation or corneous spinulation (Fig. 1A-D). The reason is that for larger samples a wider field of view invariantly compromises resolution. Hermit crabs are also a challenging taxon to study using μCT scanning, as a vast proportion of the body consists of soft tissue. With the lowest resolution of the presented scans, the data of *G. poorei* were only usable for visualizations of the well-calcified areas of the exoskeleton, like the chelipeds (Fig. 1A-B). Also, although being a fresh sample and not preserved in ethanol, the left second antenna moved slightly during scanning process as the sample was drying (Fig. 1A). We found that scanning specimens (particularly larger ones) in an airtight container

to prevent them from drying out exacerbated the problem of having to move the sample further away from the x-ray source of the scanner, causing significant loss of resolution. Therefore, and because hermit crabs have many joints and flexible soft parts that are both prone to movements, the better compromise was to keep the scanning time short when the fresh samples were scanned while exposed to air.

The scans of *P. atkinsinae* are of better quality than the one of *G. poorei*: both were scanned with settings to keep the scanning time below 30 min. The quality of the latter allowed the visualization of some soft tissue, like the pleon (Fig. 1C), and even some information on the gills are retrievable from the scan of the female (Fig. 1D). Further overcoming the problem of drying samples and sample movements during the scans, scanning the holotype of *P. fraserorum* in ethanol resulted in a clean, high-resolution surface scan (Fig. 2A). Nevertheless, the decreased density difference between the sample and the surrounding medium hindered detection of fine, soft structures. It particularly 'removed' all setation, although some setae are visible in the highest resolution scan of the left cheliped, but only if the brightness contrast threshold in the 3D rendering is set very low. However, it is then almost impossible to separate the sample from 'noise' like the mounting material and to get a clean image (Fig. 2C).

Mostly because long-term effects on tissue by staining agents remain unknown [11], we refrained from contrast enhancement techniques for these rare or type specimen scans. Moreover, a test scan using iodine as staining agent did not result in significantly better image contrast on taxonomically important features (scan not included in this dataset). Secondly, after sample preparation and the iodine-staining test scan, we noticed that the eggs of the stained and scanned ovigerous female started to fall off the pleopods more easily. Although setation was difficult to visualize in the two separate scans of the holotype of *P. fraserorum*, particularly the scan of the left cheliped reveals exceptional quality of detail. It is, for example, possible to detect the exact position of each seta as a depression from which each seta arises on the exoskeleton surface (Fig. 2D-C). Combined, the two scans of this holotypic specimen show great detail, but predominantly of calcified body parts only. Scanned in the same way while being submerged in ethanol, the scan data of *C. macrothrix* are of high quality as well.

In contrast to the in-ethanol scans of *P. fraserorum*, they also reveal a number of details that include soft tissue. For example, the very thick setae, as alluded to in the species name of *C. macrothrix*, are easily visible (Fig. 1E-F), highlighting that visualizations of soft tissue depend on sample characteristics.

Overall, the two scans (holotype *D. albimanus* and female paratype *P. fraserorum*), which were performed with the samples wrapped in parafilm, potentially show the most detail of all scans (Fig. 2 B, E-F). Not being placed in a container, the samples could be mounted extremely close to the x-ray source of the scanner, which significantly improved resolution of the scans. From these two scans it is therefore possible to retrieve information on many soft tissue parts, including the pleon and eggs attached to the pleopods, small corneous spinules, and setation. However, the quality of the data is still not sufficient, for example, to study the taxonomically highly important gills. Furthermore, this sample preparation had a disadvantage in that, wherever parafilm touched the specimen, this created unwanted scanning surface artefacts that are difficult to eliminate in the visualizations. Such scanning artefacts resulting from wrapping material can, for example, be found around the shield of the female *P. fraserorum* (Fig. 2B), as well as on the left cheliped of *D. albimanus* (Fig. 2E-F), but also in the scan of *C. macrothrix* scanned in a small container in ethanol (Fig. 1 E-F). Furthermore, in the scan of *D. albimanus*, the parafilm also increased beam-hardening effects on the edge of the scan and ventrally of the specimen, but these are easily removed in the visualization software. Lastly, the female paratype of *P. fraserorum* has a slightly broken right cheliped, which might have been damaged during collection. The cracks in the exoskeleton of the carpus are visible in the scan (not pictured in the figures), but are not a result of the scanning and are rather derived from the damaged sample. However, all major specimen characters can still be studied in great detail.

In conclusion, quality of each scan in this dataset varied and is dependent on sample characteristics and scanning protocol. Due to a lower resolution from a wider field of view, the scans of the larger specimens show good surface details of the calcified body parts, but insufficiently depict information on small features. In contrast, the scans of the smaller specimens show better details including some soft tissue, mainly because their small size allowed scanning at a

much higher resolution. Protocol optimization for future studies includes the use of the smallest possible container in which the sample can be placed (in air, but without touching container and mounting material), and which can be mounted as close to the x-ray sources of the scanner as possible. However, in this study, a good compromise was found that secured high scanning quality while keeping the effort of data collection reasonable.

Re-use potential

While the presented dataset can be used for morphological studies in general, any such research attempt would lie within the limitation of using information derived from the calcified parts of the specimens. As mentioned above, particularly the scans of the larger specimens of *G. poorei* and *P. atkinsonae* are too low in resolution to include analyses of soft tissue. The female paratype of *P. atkinsonae* is also still located in a carcinoecium shell of an anthozoan species (probably *Epizoanthus* spp.) that would have to be virtually removed prior to analysis. However, the scan can also be used to study the zooanthid shell itself, which remains a poorly known structure. Some soft tissue information will be retrievable from the higher resolution scans of the *D. albimanus* and *P. fraserorum* specimens that allowed narrow field of view of the scanner, as well as of the medium-sized *C. macrothix*. This dataset was not designed for the analyses of internal anatomy and contains no or little information on internal organs. Instead, the value of the presented scans lies in the potential to download a 3D virtual copy of museum specimens that otherwise would have to be loaned. Shipment of specimens involves significant cost and effort, as well as the potential risk of damage, or even the entire loss of a specimen, while this dataset is freely available for download and can also be examined by an unlimited number of people simultaneously. At the same time, it serves as an insurance policy should the original specimens ever get damaged or lost.

Using this dataset, researchers who want to validate a species and examine the specimens for the comparison to other taxa are provided with a 3D virtual, interactive view that allows deriving character information of some soft tissue and a suite of calcified characters. These are mainly the shield and cephalic

appendages, the chelipeds, the pereopods, as well as the uropods and the telson. Experts on hermit crab taxonomy might object to the absence of information on important soft structures, such as the gills. Nevertheless, even if the provided scans do not show all the important characters that are currently used in hermit crab taxonomy, they do show many such characters like the 3D shape of the chelipeds and pereopods, in an exceptional way. Furthermore, the digital third dimension allows for internal character examination, even of type material.

Availability of supporting data

The presented dataset is deposited in the GigaScience Database repository [..]. In a previously published dataset on brittlestars, which is so far the only other available μCT dataset of South African invertebrates, we provided the full raw data as x-ray projection images from the scanner [12]. We now realize that users would not use the projections from the scanner and that these data make the dataset unnecessarily large. Therefore, for the dataset here, each scan contains the reconstructed stack slice images both available as 16-bit tiff, and to compress the file sizes further also as 8-bit jpg stacks. The stack slice images comprise the volume data of the region of interest and can be read by any 3D visualization software package; slice images can also simply be viewed in any image viewer program. In addition, each scan folder contains a PCA file with the respective scan settings.

Declarations

List of abbreviations

3D: Three dimensional, CT: Computed tomography, μCT: Micro computed tomography

Ethics approval and consent to participate

Sampling and the handling of all biological samples were carried out under the University of Cape Town Science Faculty Animal Ethics Committee approval, protocol number 2014/DC1/CLG.

Competing interests

The authors declare that they have no competing interests.

Funding

Funding for this project was made available through a grant to CLG from the SeaKeys Project, managed by the South African National Diversity Institute (SANBI) and financed through the National Research Foundation (NRF), as well as through a doctoral research scholarship to JL from the University of Cape Town (UCT).

Authors' contributions

This study forms part of JLs PhD project on the biodiversity of South African hermit crabs. JL designed the study and wrote the manuscript. ADP prepared the data for upload and contributed to the writing of the technical section. CLG edited the manuscript. All authors read and approved the final manuscript.

Authors' information

JL is a PhD student at the University of Cape Town. Under the supervision of CLG he studies the biodiversity of hermit crabs in South Africa, but has a broad interest in marine invertebrates and in crustacean biology in particular.

As a trained physicist, ADP manages the CT Scanner section of the Central Analytical Facility, Stellenbosch University. His broad research interest includes the biological applications of CT scanning and bio-inspired engineering.

CLG is Emeritus Professor at the University of Cape Town. His main current research interest is the documentation of South African marine biodiversity.

Acknowledgements

We would like to thank Stephan G. Le Roux for help with the scans and data processing. Sarah Faulwetter is thanked for an idea-sparking E-mail discussion that refreshed the motivation of publishing our μCT data.

References

1. Faulwetter S, Dailianis T, Vasileiadou K, Kouratoras M. Can micro-CT become

an essential tool for the 21st century taxonomist? An evaluation using marine polychaetes. Microsc Anal. 2014;11:9–12.

2. Akkari N, Enghoff H, Metscher BD. A new dimension in documenting new species: high-detail imaging for myriapod taxonomy and first 3D cybertype of a new millipede species (Diplopoda, Julida, Julidae). PLoS One. 2015;e0135243.

3. Garcia FH, Fischer G, Liu C, Audisio TL, Alpert GD, Fisher BL, et al. X-Ray microtomography for ant taxonomy: an exploration and case study with two new Terataner (Hymenoptera, Formicidae, Myrmicinae) species from Madagascar. PLoS One. 2017;12:e0172641.

4. McLaughlin PA, Komai T, Lemaitre R, Rahayu DL. Annotated checklist of anomuran decapod crustaceans of the world (exclusive of the Kiwaoidae and families Chirostylidae and Galatheidae of the Galatheoidae) Part I - Lithodoidea, Lomisoidea and Paguroidea. Raffels Bull Zool. 2010;23:5–107.

5. Landschoff J, Lemaitre R. Differentiation of three common deep-water hermit crabs (Crustacea, Decapoda, Anomura, Parapaguridae) from the South African demersal abundance surveys, including the description of a new species of *Paragiopagurus* Lemaitre, 1996. Zookeys. 2017;676:21–45.

6. Landschoff J, Rahayu DL. A new species of hermit crab (Crustacea: Decapoda: Diogenidae) from the coast of KwaZulu-Natal, South Africa. Zootaxa (submitted).

7. Landschoff J, Komai T, Du Plessis A, Gouws G, Griffiths CL. MicroCT imaging applied to description of a new species of *Pagurus* Fabricius, 1775 (Crustacea: Decapoda: Anomura: Paguridae), with selection of three-dimensional type data. PLOS ONE (ready to be submitted).

8. Landschoff J, Lemaitre R. Crossing the Indian Ocean: a range extension for *Goreopagurus poorei* Lemaitre & McLaughlin, 2003 (Crustacea: Decapoda: Paguridae). Zootaxa (in press).

9. du Plessis A, le Roux SG, Guelpa A. The CT Scanner Facility at Stellenbosch University: an open access X-ray computed tomography laboratory. Nucl Instr Meth Phys Res B. 2016;384:42–9.

10. du Plessis A, Broeckhoven C, Guelpa A, Gerhard SG. Laboratory X-ray micro-computed tomography: a user guideline for biological samples. Gigascience. 2017;1–27.

11. Faulwetter S, Vasileiadou A, Kouratoras M, Dailianis T, Arvanitidis C. Micro-

computed tomography: introducing new dimensions to taxonomy. Zookeys. 2013;263:1–45.

12. Landschoff J, Du Plessis A, Griffiths CL. A dataset describing brooding in three species of South African brittle stars, comprising seven high-resolution, micro X-ray computed tomography scans. GigaScience. 2015;4:52.

Fig. 1 Micro CT scanning images (two-dimensional) and combination of surface and volume reconstructions (three-dimensional) of medium-sized hermit crabs (50-70 mm total length). A-B *Goreopagurus poorei*, male 4.5 mm SL (USNM 1292090); C *Paragiopagurus atkinsonae*, male holotype 7.0 mm SL (USNH 1292083); D *Paragiopagurus atkinsonae*, female paratype (in carcinoecium shell, SAMC MB-A066812); E-F *Cancellus macrothrix*, male/female 9.0 mm SL (SAMC MB-A066204).

Fig. 2 Micro CT scanning images (two-dimensional) and combination of surface and volume reconstructions (three-dimensional) of small-sized hermit crabs (20 mm total length). A *Pagurus fraserorum*, male holotype 2.7 mm SL (SAMC MB-A066790); B *Pagurus fraserorum*, ovigerous female paratype 2.4 mm SL (SAMC MB-A066770); C-D left cheliped of same as A; E-F *Diogenes albimanus*, ovigerous female paratype 2.0 mm SL (SAMC MB-A066353).

Click here to download Figure Fig 1.tif

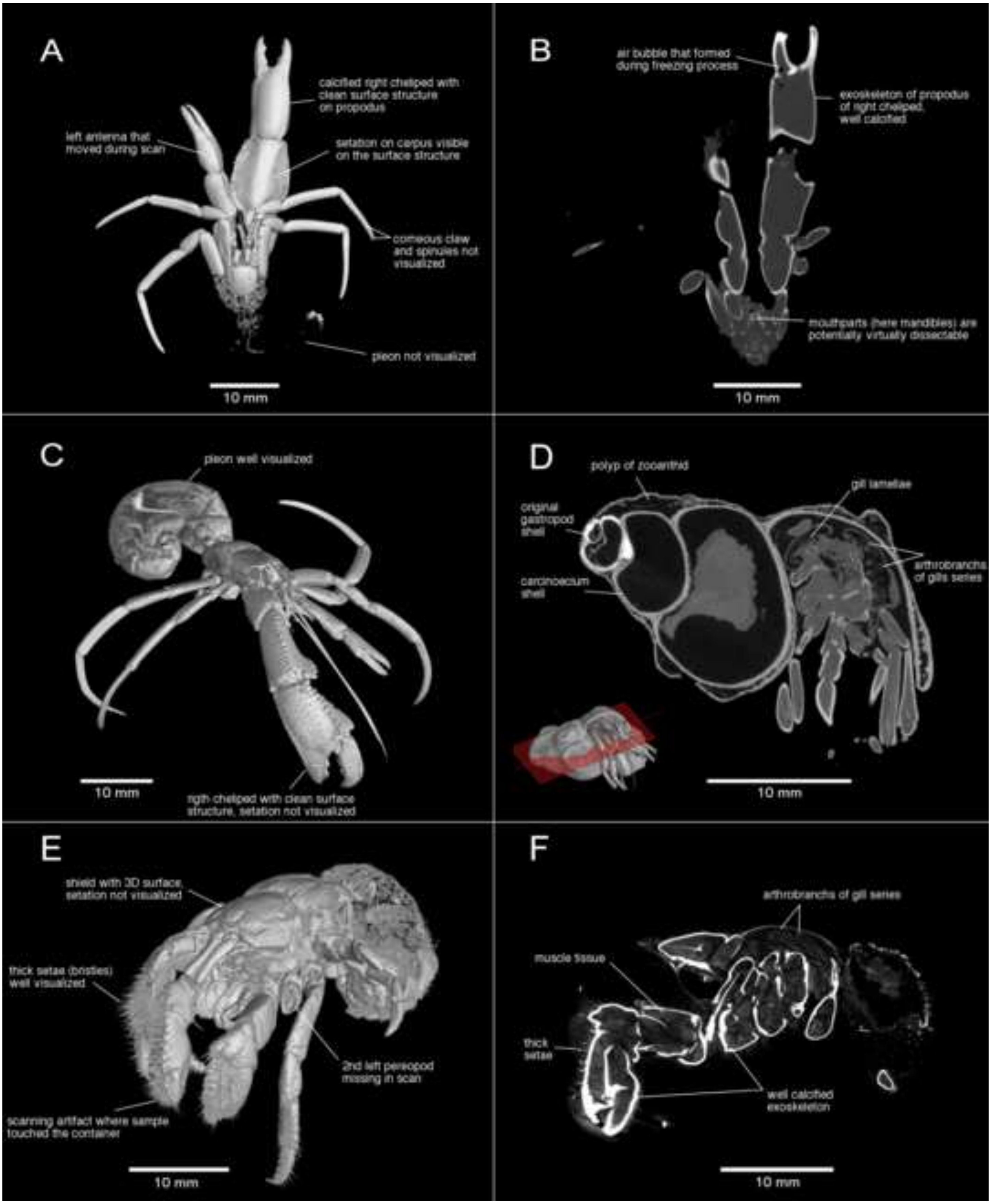

Click here to download Figure Fig 2.tif

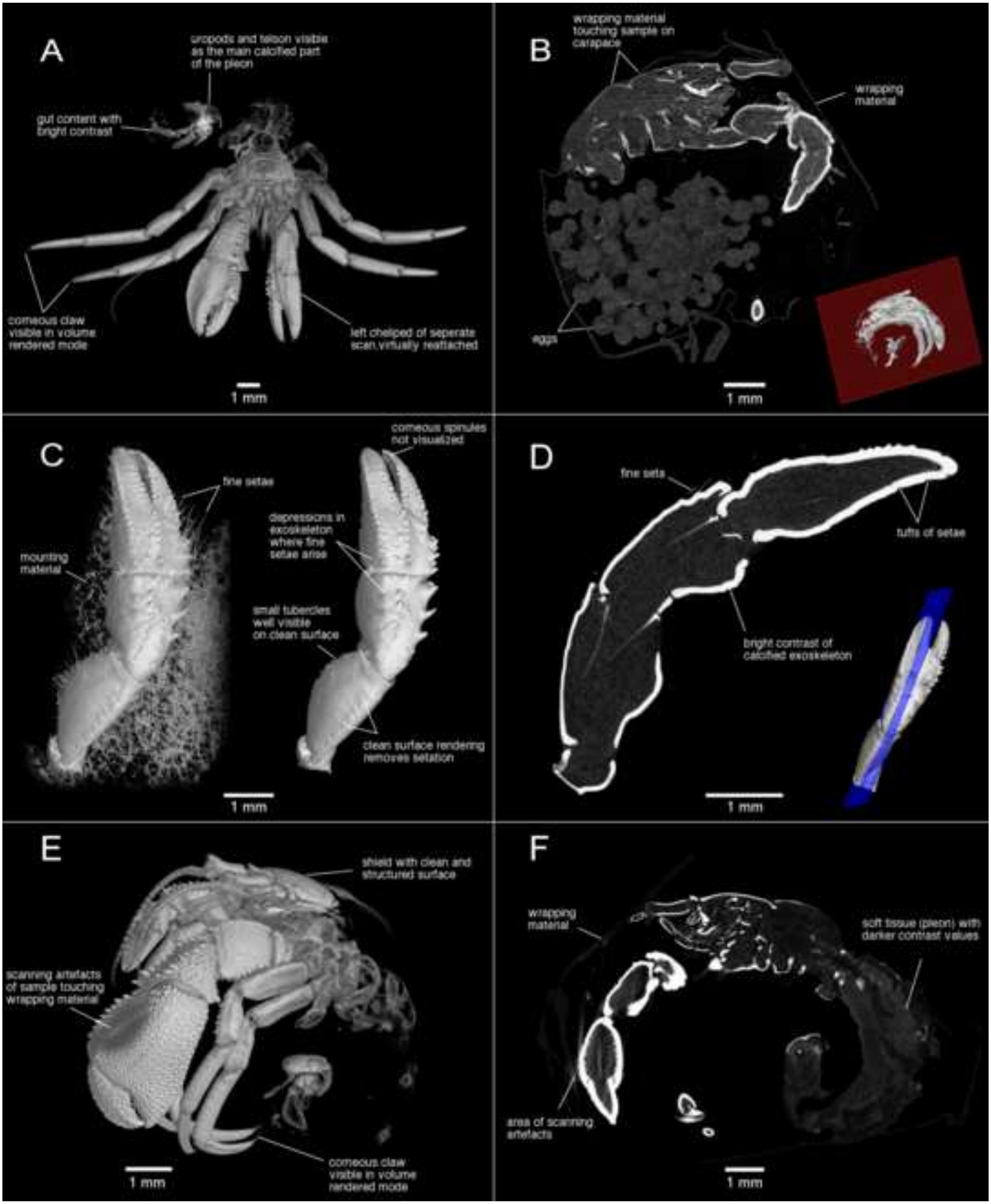

Personal Cover

Click here to download Personal Cover Cancellus_macrothrix.jpg

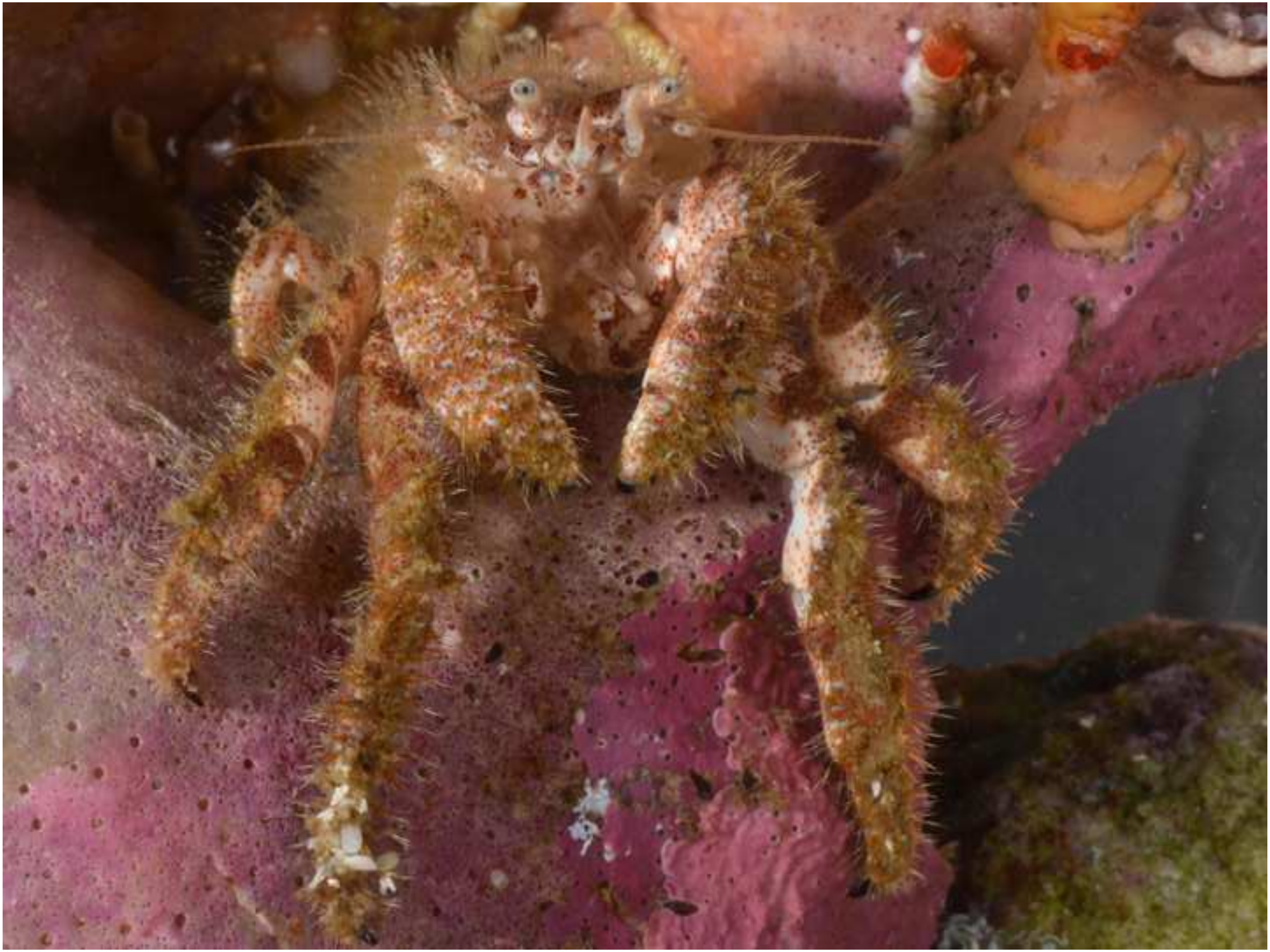

Supplement: GIGA-D-17-00200_Original_Submission.pdf [file giy022_giga-d-17-00200_original_submission.pdf]
