# Supplementary material for: A micro X-ray computed tomography dataset of South African hermit crabs (Crustacea: Decapoda: Anomura: Paguroidea) containing scans of two rare specimens and three recently described species
Source: Gigascience. 2018 Mar 14;7(4):giy022. doi: 10.1093/gigascience/giy022 (PMC5890486; doi:10.1093/gigascience/giy022)
Supplement: Response_to_Reviewer_Comments_Original_Submission.pdf [file giy022_response_to_reviewer_comments_original_submission.pdf]

Author's Response To Reviewer Comments

Dear Scott Edmunds,

Thank you very much for sending us the decision on our manuscript. We are very pleased with the outcome of the reviewing process and found both reviewers comments most useful to improve the manuscript.

We would also like to apologise for the inconvenience with the data upload and corrupt files, and my co-author Anton du Plessis has already updated and commented on this matter via E-mail. Furthermore, STL files have been created for all files, so that I believe that we are on the right track towards the goal of publishing the datanote. Please let us know if any problems remain and we will find the best way to sort them out.

With regards to the the manuscript itself I have gone over the text and made changes according to all suggestions (please refer to the track changes in the resubmitted manuscript). One main critique was with regards to the table, which I have now updated and provided the requested information.

Otherwise I have followed all suggestions of the reviewers with one exception. Reviewer 2 suggested a figure label abbreviation system to declutter the figures. While I think that this is not an entirely unvalid point, at the same time I think that this is also a matter of taste. Personally, when reading papers I prefer to have as much information in the figures themselves, so that I don't have to look for a list of abbreviations in the figure caption. However, I do acknowledge that overloaded figures are also not great and that labels should of course be large enough to be read. I have therefore shortened a few of the labels and also corrected some additional mistakes in them, but for now left the general figure design as it was. It would be great to have your opinion as the editor as to whether the labels are large enough in the current form. If so, I would like to keep them as such, but am also happy to work on them a bit more. I would like to refrain from an abbreviation list if possible.

Lastly, I am still waiting to hear from the other manuscript submissions (one Zootaxa, and one PlosOne). I will keep you updated as soon as I hear from them. I hope that this will happen soon in order to be able to use the correct scientific names in the datanote. If not, please be aware that we will not be able to use the two names of P. fraserorum and D. albimanus and would have to lable them as "sp." in both the manuscript as well as in the data, as these species have not yet been officially described! Lets hope for the best.

Please do not hesitate to contact me should any questions remain.

On behalf of all authors.
Kind regards,
Jannes Landschoff
