# Supplementary material for: A micro X-ray computed tomography dataset of South African hermit crabs (Crustacea: Decapoda: Anomura: Paguroidea) containing scans of two rare specimens and three recently described species
Source: Gigascience. 2018 Mar 14;7(4):giy022. doi: 10.1093/gigascience/giy022 (PMC5890486; doi:10.1093/gigascience/giy022)
Supplement: Response_to_Reviewer_Comments_Revision_1.pdf [file giy022_response_to_reviewer_comments_revision_1.pdf]

Author's Response To Reviewer Comments

Dear Scott Edmunds,

I have now gone over the last edits and suggestions. I deleted the unpublished paper from the reference list, and also added the last sentences in the data availability section.

Please also see my comments and track changes in the revised version.

I am looking forward to hearing from you and the production team.

On behalf of all authors,
Jannes Landschoff
