# Supplementary material for: A micro X-ray computed tomography dataset of South African hermit crabs (Crustacea: Decapoda: Anomura: Paguroidea) containing scans of two rare specimens and three recently described species
Source: Gigascience. 2018 Mar 14;7(4):giy022. doi: 10.1093/gigascience/giy022 (PMC5890486; doi:10.1093/gigascience/giy022)
Supplement: Reviewer_1_Report_(Original_Submission) -- Chris Armit — 01 Sep 2017 Reviewed [file giy022_reviewer_1_report_(original_submission).pdf]

Reviewer Report

Title: A micro X-ray computed tomography dataset of South African hermit crabs (Crustacea: Decapoda: Anomura: Paguroidea), containing scans of two rare specimens and of three recently described species

Version: Original Submission **Date:** 01 Sep 2017

Reviewer name: Chris Armit

Reviewer Comments to Author:

This is an excellent descriptive paper that describes a variety of CT scanning protocols that were used to image a diverse set of hermit crab specimens. The findings are supported by high-quality CT reconstructions (16-bit TIFF format image stack) that are made publicly available. The authors additionally provide 8-bit versions of the 3D reconstructions (JPEG format image stack), and a surface-rendered 3D image (STL format) of one of the specimens. The authors describe the utility of the CT imaging approach in the study of calcified parts. In addition they highlight the limitation of this approach in, for example, the morphological study of gills and setae. The descriptions are meticulous and some of the observations, for example, the advantages of CT imaging in ethanol, are of wider interest for the imaging of invertebrate specimens.

Minor comments

A voxel size table is provided in the Supplementary data. It would be beneficial to include the units of measurement (e.g. mm) in this table. Furthermore, although there is mention of isotropic voxel resolution in the main body of the text (i.e. the dimensions are equal in all three directions) it would be additionally useful to describe this in the table.

There are a small number of type errors that are listed below:

Abstract (as embedded in the cover page)
"loosing" - should be "losing"

Line 74
"increasing frequently" - consider revising

Line 100-101
"physical museum voucher" - consider revising

Line 150
"zooanthid" - should be "zoanthid"

Line 182

"du Plessis et al." - is this one of the listed references?

Line 302
"zooanthid" - should be "zoanthid"

Table 1
"zooanthid" - should be "zoanthid"

Level of Interest

Please indicate how interesting you found the manuscript: An article of importance in its field

Quality of Written English

Please indicate the quality of language in the manuscript: Acceptable

Declaration of Competing Interests

Please complete a declaration of competing interests, considering the following questions:

- Have you in the past five years received reimbursements, fees, funding, or salary from an organisation that may in any way gain or lose financially from the publication of this manuscript, either now or in the future?
- Do you hold any stocks or shares in an organisation that may in any way gain or lose financially from the publication of this manuscript, either now or in the future?
- Do you hold or are you currently applying for any patents relating to the content of the manuscript?
- Have you received reimbursements, fees, funding, or salary from an organization that holds or has applied for patents relating to the content of the manuscript?
- Do you have any other financial competing interests?
- Do you have any non-financial competing interests in relation to this paper?

If you can answer no to all of the above, write 'I declare that I have no competing interests' below. If your reply is yes to any, please give details below.

I declare that I have no competing interests.

I agree to the open peer review policy of the journal. I understand that my name will be included on my report to the authors and, if the manuscript is accepted for publication, my named report including any attachments I upload will be posted on the website along with the authors' responses. I agree for my report to be made available under an Open Access Creative Commons CC-BY license (http://creativecommons.org/licenses/by/4.0/). I understand that any comments which I do not wish to be included in my named report can be included as confidential comments to the editors, which will not be published.

I agree to the open peer review policy of the journal

To further support our reviewers, we have joined with Publons, where you can gain additional credit to further highlight your hard work (see: https://publons.com/journal/530/gigascience). On publication of this paper, your review will be automatically added to Publons, you can then choose whether or not to claim your Publons credit. I understand this statement.

Yes
