# Supplementary material for: A micro X-ray computed tomography dataset of South African hermit crabs (Crustacea: Decapoda: Anomura: Paguroidea) containing scans of two rare specimens and three recently described species
Source: Gigascience. 2018 Mar 14;7(4):giy022. doi: 10.1093/gigascience/giy022 (PMC5890486; doi:10.1093/gigascience/giy022)
Supplement: Reviewer_2_Report_(Original_Submission) -- Stephanie Köhnk — 10 Sep 2017 Reviewed [file giy022_reviewer_2_report_(original_submission).pdf]

Reviewer Report

Title: A micro X-ray computed tomography dataset of South African hermit crabs (Crustacea: Decapoda: Anomura: Paguroidea), containing scans of two rare specimens and of three recently described species

Version: Original Submission **Date:** 10 Sep 2017

Reviewer name: Stephanie Köhnk

Reviewer Comments to Author:

This paper provides important datasets of five species of hermit crabs. Especially the sharing of 3D data of type specimens is of high value for taxonomists and morphologists.
The manuscript is clearly written and shows some good sample pictures.

Unfortunately, there was a serious issue with the data made available, as detailed below.

Major Compulsory Revisions

The zip files for the following species were corrupted and therefore not available for evaluation: Cancellus macrothrix, Pagurus fraserorum (m holotype and f paratype). Especially the dataset of the male P. fraserorum would be interesting, since it is a combination of two datasets with different resolutions.

Minor Essential Revisions

(1) I suggest to declutter the labels on both figures. The texts are very long and therefore rather small on the images. Please think of an abbreviation system (e.g. ab instead of "air bubble that formed during freezing process") and move the longer descriptions into the figure captions/text.

(2) It would be nice to include the fixation and sample preparation method used during scanning into the table, so it is easier to see which specimen was frozen/in ethanol/wrapped in parafilm at a single glance.

(3) In the abstract, the paragraph findings starts with the following sentence (line 39): "Micro computed tomography (µCT) scan data of five hermit crab species from South Africa, two of rare specimens, three of holotypic specimens, and two of selected paratypes, depicted main identification characters of calcified body parts." This sentence is unclear, since there are five species but seven specimens and it is unclear which each label (rare, type). I suggest rephrasing: "Micro computed tomography (µCT) scan data of five hermit crab species from South Africa, including rare specimens and type material, depicted main identification characters of calcified body parts."

(4) line 245: I find the use of "thick setae" as an example for soft tissue slightly irritating. Setae are

covered in cuticle and even though flexible, they are still part of the hard structure that is the exoskeleton. Maybe rephrase to "details that include soft or fine structures"?

Please see further minor comments along the manuscript.

Level of Interest

Please indicate how interesting you found the manuscript: An article of importance in its field

Quality of Written English

Please indicate the quality of language in the manuscript: Acceptable

Declaration of Competing Interests

Please complete a declaration of competing interests, considering the following questions:

- Have you in the past five years received reimbursements, fees, funding, or salary from an organisation that may in any way gain or lose financially from the publication of this manuscript, either now or in the future?
- Do you hold any stocks or shares in an organisation that may in any way gain or lose financially from the publication of this manuscript, either now or in the future?
- Do you hold or are you currently applying for any patents relating to the content of the manuscript?
- Have you received reimbursements, fees, funding, or salary from an organization that holds or has applied for patents relating to the content of the manuscript?
- Do you have any other financial competing interests?
- Do you have any non-financial competing interests in relation to this paper?

If you can answer no to all of the above, write 'I declare that I have no competing interests' below. If your reply is yes to any, please give details below.

I declare that I have no competing interests.

I agree to the open peer review policy of the journal. I understand that my name will be included on my report to the authors and, if the manuscript is accepted for publication, my named report including any attachments I upload will be posted on the website along with the authors' responses. I agree for my report to be made available under an Open Access Creative Commons CC-BY license (http://creativecommons.org/licenses/by/4.0/). I understand that any comments which I do not wish to

be included in my named report can be included as confidential comments to the editors, which will not be published.

I agree to the open peer review policy of the journal

To further support our reviewers, we have joined with Publons, where you can gain additional credit to further highlight your hard work (see: https://publons.com/journal/530/gigascience). On publication of this paper, your review will be automatically added to Publons, you can then choose whether or not to claim your Publons credit. I understand this statement.

Yes
